# Characterization and Biofilm Inhibition of Multidrug-Resistant *Acinetobacter baumannii* Isolates

**DOI:** 10.1155/ijm/5749982

**Published:** 2024-12-28

**Authors:** Poonam Yadav, Sreska Shrestha, Deepak Basyal, Ananda Tiwari, Ranjit Sah, Anil Kumar Sah, Bishal Yadav, Mark Willcox, Shyam Kumar Mishra

**Affiliations:** ^1^Department of Microbiology, Chitwan Medical College, Tribhuvan University, Chitwan, Nepal; ^2^Department of Microbiology, National Public Health Laboratory, Ministry of Health and Population, Kathmandu, Nepal; ^3^Department of Pharmacy, Maharajgunj Medical Campus, Institute of Medicine, Tribhuvan University, Kathmandu, Nepal; ^4^Department of Pharmacognosy, Keimyung University, Daegu, Republic of Korea; ^5^Department of Health Security, Expert Microbiology Research Unit, Finnish Institute for Health and Welfare, Helsinki, Finland; ^6^Department of Microbiology, Tribhuvan University Teaching Hospital, Kathmandu, Nepal; ^7^Department of Molecular Biology, Annapurna Research Center, Maitighar, Kathmandu, Nepal; ^8^Department of General Practice and Emergency Medicine, Chitwan Medical College, Tribhuvan University, Chitwan, Nepal; ^9^School of Optometry and Vision Science, Faculty of Medicine and Health, University of New South Wales, Sydney, Australia

**Keywords:** *A. baumannii*, biofilm inhibition, biofilm production, biofilm-related gene(s), essential oils, multidrug-resistant (MDR)

## Abstract

Multidrug-resistant (MDR) *Acinetobacter baumannii* poses a significant therapeutic challenge due to its resistance to multiple antibiotics and its ability to form biofilm. This study aimed to characterize MDR *A. baumannii* isolates for their biofilm-forming capabilities and the presence of common biofilm-related genes at a tertiary care university hospital in Nepal. In addition, it assessed the efficacy of various compounds, particularly essential oils, in inhibiting biofilm formation. Identification and antibiotic sensitivity testing of *A. baumannii* isolates from clinical specimens were conducted according to the guidelines of the American Society for Microbiology. Isolates were screened for motility profiles, biofilm production in a microtiter plate assay, and the presence of biofilm-related gene(s) by conventional polymerase chain reaction. The ability of cinnamaldehyde, ethylenediaminetetraacetic acid (EDTA), Tween 80, amino acids (glycine and glutamic acid), and natural plant extracts to inhibit biofilm formation was also tested using the microtiter plate system. Out of the total 200 *A. baumannii* isolates, 195 were MDR, with 192 able to produce biofilms. Among them, 83.1% were strong biofilm producers. In this study, 42.0% and 66.2% of the isolates exhibited twitching motility and surface-associated motility, respectively. Thirty MDR *A. baumannii* isolates from medical devices contained biofilm-related genes *csuE, ompA, bap,* and *bla*_PER−1_, in 90.0%, 53.3%, 46.6%, and 26.6% of strains, respectively. Cinnamaldehyde (0.875 mg/mL) was the most effective compound, inhibiting biofilm formation by 77.3%, followed by ethanolic extract of onion (77.2%), 0.5% Tween 80 (76.8%), and essential oil of ginger (70.8%). The majority of *A. baumannii* clinical isolates were strong biofilm producers and often possessed the biofilm-related genes *csuE* and *ompA*. Essential oils at 200 mg/L, along with Tween 80, were the most effective (≥ 67%) at inhibiting the formation of biofilms. These findings help to understand biofilm production and provide valuable insights into MDR *A. baumannii* isolates in this clinical setting.

## 1. Introduction

The World Health Organization has categorized carbapenem-resistant *Acinetobacter baumannii* as the foremost critical priority pathogen, urgently requiring new therapeutics [1, 2]. *A. baumannii* is often associated with device-associated infections, e.g., ventilator-associated pneumonia (VAP), central line–associated bloodstream infection (CLABSI), and catheter-associated urinary tract infection (CAUTI), as well as traumatic wound injury especially in patients of intensive care units (ICUs) [3, 4]. Crude mortality rates associated with multidrug resistant (MDR) *A. baumannii* range from 26% to 68% [5]. Apart from acquiring antibiotic resistance, *A. baumannii* can develop antibiotic tolerance through biofilm formation which is mediated by autoinducers called acyl homoserine lactones (AHLs). These AHLs are also involved in the induction of virulence factors and antibiotic resistance via plasmid transfer [6]. Biofilm-encased cells can persist on hospital surfaces, posing a risk of outbreaks, and device-associated MDR infections among susceptible patients have made this bacterium an important pathogen [7, 8].

*A. baumannii* displays twitching motility that allows the organism to spread rapidly on semisolid and certain abiotic surfaces rather than gliding, sliding, swimming, or swarming motility [9]. Twitching motility is mediated by Type IV pili by the action of extension and retraction of the pili [10]. Bacterial motility helps cells spread across surfaces and from specific infection sites [11]. Further, twitching motility is involved in the adherence of *A. baumannii* to the surfaces and in the production of biofilm [12]. Many genes are involved in the formation of biofilms, including outer membrane protein A (*OmpA*), biofilm-associated protein (*Bap*), beta-lactamase PER-1 (*bla*PER-1), and chaperone-usher pili *(csuE)* gene [13, 14]. *OmpA* gene is a prominent porin in *A. baumannii* and contributes to drug resistance, adhesion to epithelial cells, and biofilm formation [15]. Bap is a surface-exposed, highly divergent protein that is secreted via a Type I secretion system, mediates water channel formation within biofilm, and helps in their maturation [16]. *bla*PER-1 gene is associated with increased biofilm formation and increased bacterial attachment to the abiotic surfaces and human epithelial cells [17]. The Csu assembly system, composed of pilin subunits CsuA/B, CsuA, CsuB, and CsuE and transport proteins CsuC and CsuD, is highly conserved in biofilm-forming isolates and critical for adherence to abiotic surfaces [10].

Plant extracts from leaves, stems, and roots are rich in a wide variety of secondary metabolites such as tannins, alkaloids, phenolic compounds, and flavonoids, known for their in vitro antibiofilm properties [18]. The antibiofilm effects of natural products include suppression of cell adhesion and attachment, the inhibition of formation of the polymer matrix that encases cells, and decreasing virulence factor production, thereby blocking quorum sensing (QS) network and biofilm development [19]. The rhizome of ginger (*Zingiber officinale*) is rich in secondary metabolites such as phenolic compounds, volatile sesquiterpenes, and monoterpenoids. These metabolites possess strong antioxidant, antibacterial, antifungal, anticancer, and anti-inflammatory effects [20]. Organosulfur compounds present in garlic (*Allium sativum*) are responsible for their antimicrobial activity. Its major component, allicin, is proposed to exert its antimicrobial and antibiofilm activity through multiple mechanisms, including membrane permeabilization, change in microbial gene expression, and induction of oxidative stress [21–23]. Medicinally important phenolic compounds, known as the curcuminoids, are found in the dried rhizome of turmeric (*Curcuma longa*) [24]. Curcumin exert its antibiofilm activity via attenuation of QS [25]. Essential oil (EO) of *Ageratina adenophora* (Crofton weed) contains sterols, phenolic acids, and alkaloids which are also found to have antibiofilm activity [26]. Phenolics and polyphenol extracts from the edible part of onion (*Allium cepa*) are also known for their antimicrobial and antibiofilm properties [27].

Cinnamaldehyde, a major component of cinnamon EOs, significantly reduces QS signaling, which results in reduction or prevention of EPS formation around bacterial cells [28, 29]. Ethylenediaminetetraacetic acid (EDTA) compositions are also being developed and employed for reducing biofilms in intravascular and urinary catheters and therefore represents an antibiofilm agent, which can significantly help to reduce catheter-related bloodstream infections [30]. EDTA increases the permeability of the bacterial cell wall by binding Ca^2+^ and Mg^2+^ ions that bridge the vital lipopolysaccharide (LPS) component within the outer membrane of Gram-negative bacteria, thereby destabilizing the formed biofilm [31]. The different study showed Tween 80 damages spheroplasts, which can increase the permeability of an outer membrane of Gram-negative bacteria and may destabilize their biofilms [32].

This study aimed to assess the biofilm-forming ability of MDR *A. baumannii*, to identify biofilm-related genes and to test whether plant extracts from the local surroundings could inhibit biofilm formation.

## 2. Materials and Methods

### 2.1. Study Design, Setting, Isolation, and Identification of *A. baumannii*

This hospital-based cross-sectional study was conducted in the Department of Microbiology, among the inpatients of Tribhuvan University Teaching Hospital (TUTH), Kathmandu, a tertiary care referral center with 750 beds, from March to December 2021. Different clinical specimens, including those from both device-associated (endotracheal tubes, catheter tips, and cerebrospinal shunts) and non–device-associated specimens (blood, urine, pus, wound swab, body fluids, and sputum) were processed according to the American Society for Microbiology (ASM) guidelines [33]. The specimens were inoculated onto suitable culture media (5% human blood agar, MacConkey agar, chocolate agar, and brain heart infusion (BHI) broth (HiMedia Laboratories Pvt. Ltd, India) according to their specific requirements. Identification of isolates was performed following standard microbiological techniques which involved the morphological appearance of the colonies, Gram's staining, motility test, and a battery of biochemical tests which included catalase, oxidase, oxidation-fermentation, triple sugar iron agar, citrate utilization, urease production, decarboxylation, and growth at 37°C and 44°C tests [34].

### 2.2. Antimicrobial Susceptibility Testing

After identifying *A. baumannii* isolates, their antibiotic susceptibility was determined by the Kirby–Bauer disk diffusion method on Mueller–Hinton agar, following standard procedures recommended by the CLSI 2019 guidelines [35]. The CLSI-recommended battery of antibiotics (HiMedia Laboratories Pvt. Ltd, India) was used, with *A. baumannii* ATCC 19606 serving as the quality control strain. Isolates resistant to at least one antibiotic in three different classes of first-line drugs tested were classified as MDR. Extensively drug-resistant (XDR) isolates were defined as those resistant to at least one agent in all antimicrobial categories [36].

### 2.3. Motility Detection

Luria–Bertani (LB) broth containing 0.4% or 0.8% agar was used for motility assays. For swarming motility, bacteria from overnight grown colonies were stabbed on the surface of the 0.4% semisolid medium using a sterile wooden stick to enable spread of bacteria. For twitching motility, colonies were stabbed at interphase between the bottom of the Petri dish and medium (0.8% semisolid). The agar plates were then incubated at 37°C for 48 h. For each isolate, assays were performed at least three times.

Swarming motility was considered positive if isolates showed a zone of > 10 mm around the site of inoculation. For assessing twitching motility, following incubation, the agar was removed from the plates, and the inner surface of each petri dish was stained with 0.2% crystal violet to visualize bacterial presence macroscopically. Bacteria were classified based on their twitching motility as nonmotile (< 5-mm spread from inoculation site), intermediate motility (5- to 20-mm spread), or high motility (> 20-mm spread) [37, 38].

### 2.4. Biofilm Formation Assay

Biofilm formation was detected as previously described Stepanovic et al. [39]. Briefly, 200 *μ*L of a 1/100 times dilution (in BHI broth with 1% glucose) of 0.5 McFarland adjusted bacterial suspension was placed into wells of polystyrene microtiter plates (Tarsons, Catalog No. 941296) and incubated in static conditions for 24 h at 37°C. Negative controls wells were containing sterile uninoculated BHI broth only, while *A. baumannii* type strain ATCC 19606 was used as a positive control. The test was run in triplicate. After incubation, the microtiter plates were vigorously washed in physiological saline three times to remove planktonic and loosely adhered cells. The remaining adherent bacteria were fixed with 200 μL of 99% (v/v) methanol for 15 min and then left to dry. Plates were stained with a 2% Hucker's crystal violet for 5 min and rinsed with tap water. After complete drying, 200 μL of 33% glacial acetic acid was added to dissolve the crystal violet, and the OD of the resulting solution was measured at 550 nm using an automated ELISA reader. The cutoff optical density (ODc) was defined as three standard deviations above the mean OD of the negative control: ODc = average OD of negative control + (3 × SD of negative control). ODc value is calculated for each microtiter plate separately. Strains were classified as nonbiofilm producers (OD ≤ ODc), weak biofilm producers (ODc < OD ≤ 2 × ODc), moderate biofilm producers (2 × ODc < OD ≤ 4 × ODc), or strong biofilm producers (4 × ODc < OD).

### 2.5. Detection of Gene(s) Involved in Biofilm Formation of MDR *A. baumannii* Isolates

Due to the limited funding, biofilm-related genes were studied only in isolates from medical devices. Medical device–associated infections are a critical concern in healthcare, often leading to severe complications and increased healthcare costs. By prioritizing these isolates, study can directly address these high-impact issues, optimizing the use of limited financial resources. These tests were performed at the molecular laboratory of Annapurna Research Center, Kathmandu, Nepal. Three to four isolated colonies from each biofilm-producing MDR *A. baumannii* isolates from medical devices were inoculated in 3 mL of trypticase soy broth. After overnight incubation at 37°C, genomic DNA was extracted using the cetyltrimethylammonium bromide (CTAB) method [40].

The presence of the biofilm-related genes *bap, ompA, csuE,* and *bla*_PER−1_ was assessed using PCR. The primers sequences are listed in Table 1. The concentration of each oligomer used in this study was 10 pM. The PCR was performed by using DreamTaq PCR Master Mix (Thermo Fisher Scientific), which contains Taq polymerase, dNTPs, MgCl_2_, and the appropriate buffer. Each PCR tube contained 15 μL reaction mixture composed of 10.4 μL of master mix, 0.6 μL of each forward and reverse primer solution (Macrogen, South Korea), and 4 μL of extracted DNA template. The PCR was conducted in a ProFlex PCR system and performed according to the following conditions: initial denaturation at 94°C for 5 min, then 30 cycles of denaturation (94°C, 1 min), annealing (the annealing temperatures for each gene are listed in Table 1) for 1 min, extension at 72°C for 1 min, followed by a final extension at 72°C for 5 min. PCR products were analyzed by electrophoresis in 1% agarose gel containing 2 μL ethidium bromide. DNA bands were observed under a UV transilluminator (UVITEC Cambridge). *A. baumannii* type strain ATCC 19606 was used as the producer of biofilm (control strain), and PCR buffer with no extracted DNA was used as the negative control.

### 2.6. Biofilm Inhibition Assays

Several experiments were conducted in which different natural and chemical agents were used to examine their ability to inhibit biofilm formation. More importantly, testing the plant extracts from our own local surroundings against *A. baumannii* biofilms had been the prime focus of this research, and it was the first study from Nepal. In Nepal, there are many studies conducted on biofilm formation; however, to our knowledge, no studies have been conducted looking for the efficacy of plant extracts as antibiofilm agents. The selection of specific natural products (ginger, garlic, turmeric, onion, chili, and *A. adenophora*) were based on their historical use in traditional medicine, documented antibacterial properties, and previous evidence of effectiveness against biofilm-forming pathogens. EDTA (125 mg/L), cinnamaldehyde (0.875 mg/mL), glycine (100 mM), glutamic acid (100 mM), and Tween 80 were purchased from HiMedia Laboratories Pvt. Ltd, India.

The rhizomes of turmeric and ginger, bulbs of garlic and onion, and chili peppers were purchased from the local market of Kathmandu, Nepal, and the leaves of *A. adenophora* (identified as *A. adenophora* in the Department of Pharmacy, Institute of Medicine, using standard techniques and collected from the garden of TUTH [26]. Before extraction, plants were washed with clean tap water.

The extraction of EOs from turmeric, ginger, garlic, and *A. adenophora* used a Clevenger apparatus. Fifty grams of each of the rhizomes of turmeric, ginger, the bulb of garlic, and leaves of *A. adenophora* was ground and heated in a one-liter round-bottom flask containing 500 mL water for 45 min in a Clevenger apparatus using steam distillation. Subsequently, the EO was separated from the water phase using a separatory funnel, and the resulting oils were kept in Eppendorf tubes wrapped with aluminum foil and stored at 4°C prior to further analysis. The extraction of onion and chili peppers was performed using the reflux condensation extraction method. Fifty grams each of onion and chili pepper was ground and then added to a 1-L round-bottom flask containing 200 mL of aqueous ethanol and refluxed for 2.5 h. Any remaining ethanol was evaporated in a water bath, and the remaining extracted residues were kept in an airtight container at 4°C until use. Tween 80 (0.1%) and dimethyl sulfoxide (DMSO) (5%) were used as solvents for the preparation of stock solutions of these different plant extracts. The stock solutions were further diluted to make 200 mg/L which was used as the working solution for biofilm inhibition method.

The chemical constituents in the EOs of ginger, garlic, turmeric, and *A. adenophora* were analyzed by gas chromatography–mass spectrometry (GC–MS). The GC–MS analysis was performed on a Shimadzu GC-MS-QP2010 Plus available at the Instrument Section of the Department of Plant Resources, Kathmandu. The capillary column used for the analysis was RTX-5MS (60 m × 0.32 mm × 0.25 *μ*m) with a crossbond of 5% diphenyl/95% dimethyl polysiloxane as the stationary phase. The GC analysis was performed under the following conditions: column oven temperature, 50°C; injection temperature, 250°C; ion source temperature, 250°C; interface temperature, 200°C; split injection mode with a split ratio of 80; helium with a pressure of 53.8 kPa; total gas flow, 112.3 mL/min; and column flow, 1.35 mL/min. The GC–MS system started with an initial oven temperature of 50°C for 1 min, and then, this was increased to 230°C at a rate of 3°C per 9 min. Mass spectral detection was carried out in electron ionization mode by scanning at 40–350 m/z. The total time required for analyzing a single sample was 60 min. The chemical components of the EOs were identified by comparing their mass spectral fragmentation patterns with those in the National Institute of Standard Technology Library (NIST) 2017 and Flavor and Fragrance Natural and Synthetic Compounds (FFNSC) 4.0 library and also by comparing the retention times of the components with those of the reference compounds. The percentage of each component (area %) was reported as raw percentages based on the total ion chromatogram (TIC) without standardization.

After extraction of EOs and collection of the other samples, the biofilm inhibition assay was performed on strong biofilm-producing MDR *A. baumannii* isolates. The bacterial inocula were prepared as described for biofilm formation assay [43, 44]. A total of 100 μL of bacterial growth were added to wells of polystyrene, U-bottom 96-well microplates (Tarsons, Catalog No. 941296) and incubated for 1 hour at 37°C to allow cell adhesion. After incubation, 100 μL of each plant extract or the chemical compounds were added at their previously prepared concentrations to the wells. The growth control wells contained standardized amounts of bacteria in BHI (100 μL) plus an additional aliquot of sterile BHI (100 μL) to bring the final volume to 200 μL without any antibiofilm agent. Each isolate was tested in duplicate and incubated at a temperature of 37°C for 24 h. After incubation, biofilm staining and quantification procedure was performed as previously described [45–47]. The antibiofilm activity was calculated as the percentage of inhibition ([(OD growth control − OD experimental sample)/OD growth control] × 100%) [45]. The final results were reported as the mean value of percentage inhibition of each corresponding antibiofilm agents using against MDR *A. baumannii* isolates.

### 2.7. Statistical Analysis

**Figure 1:**
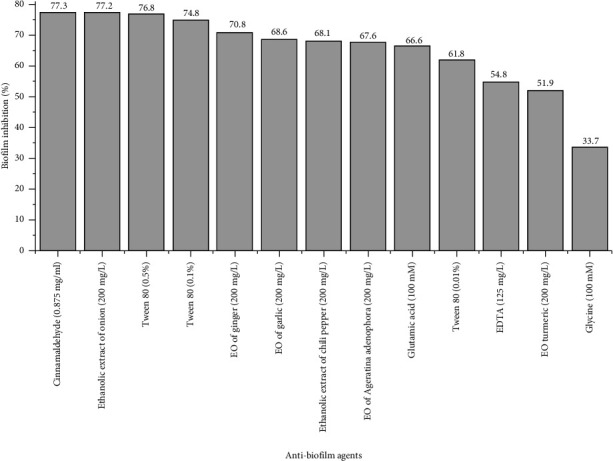
Biofilm inhibition percentage of MDR *A. baumannii* isolates by different compounds.

All data were analyzed using the SPSS Version 20 (Armonk, NY:IBM Corp.) and interpreted according to frequency distribution and percentage. Chi-square test was applied to test the significance of the relation between categorical values, and *p* value < 0.05 was considered statistically significant.  Figure 1 was made with OriginPro (OriginLab Corporation 2017).

### 2.8. Ethics Approval and Consent to Participate

The study was approved by Institutional Review Committee of Institute of Medicine, Tribhuvan University, Nepal, Reference number: 338(6-11) E^2^/077-078, and written consent was taken from patients themselves, while for patients in the Pediatrics Intensive Care Unit and Neonate Intensive Care consent was obtained from their local guardian before enrollment in the study.

## 3. Results

### 3.1. Characteristics of Isolates

In the identification of *Acinetobacter baumannii*, the following positive phenotypic characteristics were observed: the organism appeared as Gram-negative coccobacilli, was nonmotile, catalase positive, and oxidase negative. It demonstrated a nonfastidious and nonfermentative (oxidative) metabolism, producing neither gas nor hydrogen sulfide. The strain did not hydrolyze urea, but utilized citrate and was capable of hydrolyzing arginine. Additionally, it was non-hemolytic on blood agar and exhibited growth at both 37°C and 44°C. When cultured on media supplemented with 5% human blood and 0.22 M D-glucose, a characteristic brown discoloration (browning effect) was observed.

From a total of 18,343 specimens, 4249 (23.1%) showed bacterial growth, of which 200 (4.7%) were *A. baumannii.* Out of the 200 *A. baumannii* isolates, 195 (97.5%) were classified as being MDR and 180 (90.0%) were XDR. Among the total of 195 MDR *A. baumannii*, 84.6% were isolated from clinical specimens (nonmedical devices) whereas 15.4% were recovered from medical devices (Table 2). The majority of biofilm producers were recovered from general ICU (*n* = 65) followed by COVID-19 ICU (*n* = 18) and medical ICU (*n* = 12).

### 3.2. Prevalence of Antimicrobial Resistance Among MDR *A. baumannii* Isolated From Nonmedical Devices and Medical Devices

All the MDR *A. baumannii* isolates (*N* = 195) were sensitive to only antibiotics polymyxin B and colistin sulfate. Isolates from medical devices were exhibiting more resistance rate to cotrimoxazole, ciprofloxacin, levofloxacin, gentamicin, piperacillin–tazobactam except amikacin and sulbactam-containing antibiotics. The result revealed no significant difference in antibiotic resistance based on the source of *A. baumannii* isolates (*p* > 0.05) (Table 3).

### 3.3. Distribution of Biofilm-Producing Capacity and Motility of MDR *A. baumannii* Isolated From Nonmedical Devices and Medical Devices

Among the 195 MDR *A. baumannii* isolates, 192 (98.5%) could produce biofilms, with 83.1% (162 isolates) identified as strong biofilm producers. This study also showed isolates from medical devices were having only high biofilm-producing capacity while compared to isolates from nonmedical devices. Regardless of the source of isolation (tissue or medical devices), most isolates (*n* = 113, 58%) did not produce twitching motility, whereas the majority (*n* = 129, 66.2%) demonstrated swarming motility (Table 4). Motility exhibited by *A. baumannii* clinical isolates is shown in Supporting Figures (SF) 1A, 1B.

Twitching motility was more commonly seen among the isolates from sputum (data not shown). Similarly, irrespective of their resistance to any particular antibiotic, twitching motility was not seen in the majority of the isolates whereas swarming motility was observed most often (Table 5).

### 3.4. Distribution of Biofilm Formation in MDR *A. baumannii* With Respect to Twitching and Surface-Associated Motility

This study revealed that swarming motility was primarily seen in strong biofilm producers (119/162), whereas the majority did not exhibit twitching motility (100/162). The result showed there was significant difference between nature of twitching motility, surface-associated motility, and property of biofilm production of *A. baumannii* isolates (*p* < 0.05) (Table 6).

### 3.5. Genes Involved in Biofilm Formation in MDR *A. baumannii* Isolated From Medical Devices

In this study, biofilm-related gene was detected in 30 isolates of MDR *A. baumannii* isolated from medical devices which was strong biofilm producer screened by phenotypic microtiter plate method. All *A. baumannii* isolates carried at least one biofilm-related gene. Among these, the prevalence decreased in the following order: *csuE* (90.0%), *ompA* (53.3%), *bap* (46.6%), and *bla*_PER−1_ (26.6%). Examples of PCR products of the four biofilm-related genes in isolates of *A. baumannii* are shown in Supporting Figures (SF) 2A, 2B, 2C, 2D. Notably, the MDR *A. baumannii* isolates from endotracheal tubes showed the highest number of biofilm-related genes (Table 7).

### 3.6. Distribution of Biofilm-Related Genes Among Antibiotic-Resistant *A. baumannii* Isolated From Medical Devices

This study revealed that the majority of antibiotic-resistant isolates isolated from medical devices carried several biofilm-related genes (Table 8).

### 3.7. Phytochemical Constituents of the EOs of Plant Extract Identified by GC–MS Analysis

GC–MS analysis in EO of ginger revealed the presence of 31 compounds among which *β*-curcumene (14.9%), *β*-sesquiphellandrene (10.3%), geranial (9.0%), *α*-E,E-farnesene (8.3%), and camphene (6.8%) were found as the major compounds. A total of 14 compounds were identified in EO of garlic among which allitridin (35.8%), trisulfide allyl methyl (18.6%), and allyl disulfide (16.3%) were major constituents. Similarly, 27 compounds were consisted in EO of turmeric, with highest proportion being Z-*γ*-atlantone (29.0%), ar-turmerone (19.9%), and E-*γ*-atlantone (18.0%). Likewise, *α*-muurolol (12.0%), *α*-bisabolol (8.3%), cyperotundone (6.4%), bornyl acetate (5.8%), *p*-cymene (4.8%), *β*-bisabolene (4.5%), germacrene D (4.0%), and camphene (3.5%) were main constituents found in EO of *A. adenophora* among 39 identified compounds. The TIC obtained and the compounds identified through GC–MS analysis of the EOs of ginger, garlic, turmeric, and *A. adenophora* are shown in Supporting Figures SF3, SF4, SF5, and SF6, and Supporting Tables ST1, ST2, ST3, and ST4, respectively.

### 3.8. Biofilm Inhibition by Different Compounds

Biofilm inhibition assay was performed on strong biofilm-producing MDR *A. baumannii* isolates (*n* = 162). The concentration of different natural and chemical antibiofilm agents in our study was selected based on their demonstrated concentration-dependent inhibition of biofilm formation as reported in published articles [32, 43, 44, 48]. The results showed cinnamaldehyde (0.875 mg/mL) and EDTA (125 mg/L) inhibited the biofilm biomass by 77.3% and 54.8%, respectively. Different concentrations of Tween 80 (0.01%, 0.1%, and 0.5%) showed concentration-dependent inhibition of biofilm. The concentration of EOs (200 mg/L) of ginger, garlic, turmeric, *A. adenophora* prevented biofilm formation by 70.8%, 68.6%, 51.9%, and 67.6%, respectively. Ethanolic extract of onion (200 mg/L) prevented biofilm formation by 77.2% which was more as compared to that by ethanolic extract of chili pepper (68.1%). It was also observed that glutamic acid and glycine amino acids have the ability to inhibit biofilm formation in which the effect of glutamic acid on biofilm was more than that of glycine that was 66.6% as compared to glycine which showed only 33.7% of biofilm inhibition (Figure 1).

## 4. Discussion

This study reveals a high prevalence of MDR and XDR clinical isolates of *A. baumannii* at the study site over the last decade [49, 50]. The high rates of MDR *A. baumannii* observed may be attributed to factors such as emergence of bacteria with different resistance mechanisms, increased likelihood of resistance dissemination in hospital environments, absence of a robust nosocomial infection surveillance system, and suboptimal infection control practices [51].

In the present study, biofilm production was observed in nearly all isolates of MDR *A. baumannii*, with 83.1% exhibiting strong biofilm production. This finding aligns with a study conducted in a tertiary care hospital in Nepal, where 99.6% of *Acinetobacter* isolates were identified as biofilm producers and 89% of them were characterized as strong biofilm producers [52]. Strong biofilm producers are significantly associated with recurrent infections and antimicrobial resistance [53, 54].

Our study revealed that among the 195 MDR *A. baumannii* isolates, twitching motility was observed in 42.0%, while surface-associated motility was observed in 66.2%. These findings are consistent with another study where 50.0% of isolates exhibited twitching motility and 62.5% displayed surface-associated motility phenotypes [55]. Approximately 4.1% and 61.0% of MDR *A. baumannii* isolates exhibited strong twitching motility and swarming motility, respectively, along with a robust capacity for strong biofilm production. Our findings indicate that the types of biofilm producers show a significant association with the swarming and twitching motility exhibited by *A. baumannii* isolates, suggesting that these motility behaviors may play a role in biofilm formation. Notably, this study marks the first from Nepal to establish a correlation between biofilm formation and the motility traits of MDR *A. baumannii* isolates. We speculate that the greater degree of motility observed in sputum isolates may be attributed to the overexpression of Type IV pili–related genes compared to isolates from other specimens. Motility, as a trait, relies on the presence of Type IV pili, and it has been demonstrated in other bacteria that biofilm-forming cells often downregulate genes associated with motility [56].

Due to the escalating resistance of *A. baumannii* to various antibiotics, often attributed to biofilm formation, there is an urgent need to identify therapeutic strategies aimed at inhibiting biofilm formation and effectively treating established biofilms [57, 58]. In our study, treatment with EDTA (125 mg/L) resulted in a notable 54.8% inhibition in biofilm formation, consistent with findings in other studies [48, 59]. The ability of EDTA to chelate and potentiate bacterial cell walls, along with its capacity to destabilize biofilms by sequestering calcium, magnesium, zinc, and iron, positions it as a suitable agent for biofilm inhibition [60]. Similarly, cinnamaldehyde demonstrated a promising inhibition of 77.3% in average biofilm formation, surpassing the findings by Mohamed et al. [44]. In our study, Tween 80 exhibited a concentration-dependent reduction in biofilm formation, ranging from 61.8% to 76.8% as concentrations increased. Moreover, at a concentration of 100 mM, glutamic acid and glycine showed average reductions of 66.6% and 33.7%, respectively, in biofilm formation. A study from Iraq also reported significant antibiofilm activity of these compounds [43]. In addition, inhibitory effects observed against *Staphylococcus aureus*, *Pseudomonas aeruginosa*, and *Bacillus subtilis* suggest that D-amino acids may serve as a general strategy for inhibiting biofilm formation in opportunistic pathogens. D-amino acids play a role in regulating bacterial cell wall remodeling during the stationary phase, contributing to biofilm dispersal in aging bacterial communities [61–63].

The use of antibiofilm agents, capable of either inhibiting or eliminating biofilm without inducing resistance, is essential for a potential therapeutic approach to managing MDR biofilm-associated infections. As our study primarily focused on identifying alternative technologies for controlling *A. baumannii* biofilms, we turned to EOs derived from edible plants, which have been consumed by humanity since ancient times and are known for their diverse benefits. In our investigation, we employed EOs from ginger, garlic, turmeric, and *A. adenophora*, along with ethanolic extracts of onion and chili, as antibiofilm agents. Notably, to our knowledge, this study represents the first exploration in Nepal of bacterial biofilm inhibition using natural plant extracts. The GC–MS analysis of the ginger EO revealed the presence of phenolic compounds (geraniol and citronellol), volatile sesquiterpenes (bisabolene), and monoterpenoids (curcumene and *β*-sesquiphellandrene), aligning with another study [20]. In our study, the EO of ginger demonstrated an average 70.8% reduction in biofilm in *A. baumannii* isolates which is comparable to another study [64]. The EO of turmeric was found to contain secondary metabolites such as zingiberene, tumerone, ar-turmerone, curlone, and the phenolic compound curcumin, which is recognized as the most essential bioactive component among curcuminoids. Turmerone, in particular, exhibited antibiofilm properties against bacterial isolates. Our study demonstrated an average 51.9% reduction in *A. baumannii* biofilm. Similarly, Ahamad et al. showed a reduction of *A. baumannii* biofilm from 2.0170 ± 0.14863 (mean ± SD) to 0.2470 ± 0.11314 by 200 mg/L turmeric extract. In addition, Suwal et al. evaluated the antibiofilm effects of turmeric extract against *Pseudomonas aeruginosa* and *Staphylococcus aureus*, reporting inhibition of biofilm formation in *Pseudomonas aeruginosa* ranging from 26.7% to 58.5%, and in *Staphylococcus aureus* from 48.8% to 77.7%, at the concentration between 0.5 and 2 mg/ml [65]. The EO of garlic revealed major compounds such as allyl disulfide and allitridine, known for their biofilm-inhibiting properties. In our study, we observed a substantial 68.6% inhibition of biofilm in *A. baumannii* at a concentration of 200 mg/L, aligning with findings of Somrani et al., who reported a 68.0% inhibition of biofilm when garlic EO was exposed to bacteria for 1 hour at its minimum inhibitory concentration (MIC) [46]. Garlic has also been recommended in different studies as an agent to prevent wound pathogen biofilm formation when formulated as garlic ointment [66]. In addition, it can be applied on catheters to prevent catheter-associated biofilm infections [67]. Furthermore, the GC–MS analysis of the EO of *A. adenophora* identified major chemical constituents such as *α*-phellandrene, camphene, bornyl acetate, *p*-cymene, *γ*-curcumene, germacrene, and *α*-bisabolol, consistent with previous reports. These compounds have demonstrated antibacterial activity against both Gram-positive and Gram-negative bacteria [68–70]. In our study, 200 mg/L of *A. adenophora* inhibited 67.6% of biofilm formation in *A. baumannii*. Chili pepper, another focus of our study, contained secondary metabolites such as dihydrocapsaicin and luteolin, which exhibit antimicrobial and anti-QS activities against bacterial pathogens [70–72]. Another compound investigated in our study was the ethanolic extract of onion, which exhibited a notable 77.2% inhibition against *A. baumannii* biofilm. This efficacy aligns with findings demonstrating its effectiveness against *Listeria monocytogenes* biofilms as well [46]. The antibiofilm property of onion is attributed to its sulfur compounds. These compounds interact with the sulfhydryl (SH) groups of cellular proteins, forming mixed disulfides that have the potential to inflict damage upon microbial cells [73].

Numerous studies have elucidated the role of biofilm-related genes (*csuE, ompA, bap*, and *bla*_PER−1_) in *A. baumannii* in biofilm development and antibiotic resistance [42, 74]. In our study, these genes were identified in 30 strong biofilm-producing isolates of MDR *A. baumannii* obtained from medical devices. The results revealed the most prevalent gene was *csuE* (90.0%), followed by *ompA* (53.3%), *bap* (46.7%), and *bla*_PER−1_ (26.6%). Similar result was reported by Thummeepak et al., who showed a prevalence of 48% and 30.2% for *bap* and *bla*_PER−1_ genes, respectively [54]. The highest frequency of biofilm-related genes was observed in isolates from endotracheal aspirate samples. The *csuE* gene, a member of the usher–chaperone assembly system, plays a crucial role in mediating attachment and biofilm formation. The high prevalence of *csuE* in *A. baumannii* is consistent with findings from other studies [41, 42, 74, 75]. *OmpA*, another biofilm-related gene in *A. baumannii*, is likely essential for attachment to human epithelial cells, biofilm development, and antimicrobial resistance. A limitation of our study is that we did not perform 16S rRNA sequencing for confirming species identification and detecting genetic features in this study. Likewise, the activity of the compounds used for biofilm inhibition was not investigated against the planktonic bacterial cells. In addition, cytotoxicity assays and MIC tests for different antibiofilm agents were not performed, and biofilm-related genes were examined only in isolates from medical devices. Moreover, integron detection was not performed to determine the correlation between MDR and biofilm formation.

## 5. Conclusion

This study underscores the high prevalence of MDR *A. baumannii*. We found that polymyxins, followed by sulbactam-containing antibiotics, were relatively effective against MDR *A. baumannii.* Furthermore, the majority of MDR *A. baumannii* isolates were biofilm producers. Notably, isolates from medical devices exhibited significantly stronger biofilm-producing capacity than those from nonmedical devices. Our study also indicated a significant difference between biofilm formation and motility in MDR *A. baumannii*, suggesting that mechanistic investigation into motility could provide novel therapeutic strategies for controlling the persistence of this pathogen. Our investigation into nonantibiotic agents demonstrated promising antibiofilm effects against MDR *A. baumannii*. Specifically, EDTA, cinnamaldehyde, Tween 80, and amino acids (glycine and glutamic acid), natural extracts such as EOs of ginger, garlic, turmeric, *A. adenophora*, and ethanolic extracts of onion and chili exhibited significant antibiofilm activity. These agents may serve as viable natural antimicrobial alternatives for managing this pathogen. The results indicated that *csuE, ompA, bap,* and *bla*PER-1 are associated with biofilm development and likely contribute to antibiotic resistance. Understanding these mechanisms can enhance our insight into the relationship between biofilm production, antibiotic resistance, and the transmission routes of clinical isolates. These insights are valuable for designing strategies to control drug-resistant pathogens, underscoring the need for appropriate surveillance and control measures to prevent the emergence and transmission of MDR *A. baumannii* in our setting.

Further research is needed to elucidate the mechanisms underlying the antibiofilm effects of these natural and chemical agents, which represents a limitation of our study. Detailed phenotypic and genotypic characterization of MDR *A. baumannii* isolates is essential to deepen our understanding of their pathobiology and pathophysiology. Future research in Nepal should prioritize the analysis of additional biofilm-related genes in MDR *A. baumannii* to further elucidate its biofilm formation mechanisms.

## Figures and Tables

**Table 1:** The primers, primer sequence, annealing temperature, and DNA amplicon size used for the detection of biofilm-related genes.

Targeted genes (primers)	Primer sequence (5′-3′)	Annealing temperature (°C)	DNA amplicon size (bp)	References
*csuE*	FW = CATCTTCTATTTCGGTCCC RV = CGGTCTGAGCATTGGTAA	59	168	[41]

*bap*	FW = TGCTGACAGTGACGTAGAACCACA RV = TGCAACTAGTGGAATAGCAGCCCA	49	184	[41]

*ompA*	FW = GTTAAAGGCGACGTAGACG RV = CCAGTGTTATCTGTGTGACC	49	578	[41]

*bla*_PER−1_	FW = GCAACTGCTGCAATACTCGG RV = ATGTGCGACCACAGTACCAG	55	340	[42]

**Table 2:** Distribution of MDR *A. baumannii* growth in different clinical specimens.

Source of specimens	Frequency	%
Non-device associated specimens	165	84.6
Sputum	76	39.0
Pus	40	20.5
Blood	17	8.7
Body fluid	14	7.2
Urine	9	4.6
Bronchoalveolar lavage (BAL)	9	4.6
Medical device-associated specimens	30	15.4
Endotracheal tube	16	8.2
Central venous catheter	8	4.1
Urinary catheter	5	2.6
Cerebrospinal shunt	1	0.5
Total	195	100

**Table 3:** Antibiotics resistance profile of MDR *A. baumannii*

Antibiotics	Source of isolates		*p*-value
	Nonmedical devices (*N* = 165)	Medical devices (*N* = 30)	
	Resistant *n* (%)	Resistant *n* (%)	
Cotrimoxazole	157 (95.1)	30 (100)	0.611
Ciprofloxacin	163 (98.8)	30 (100)	1.000
Levofloxacin	152 (92.1)	29 (96.7)	0.700
Gentamicin	160 (97.0)	30 (100)	1.000
Amikacin	157 (95.1)	27 (90.0)	0.380
Ceftazidime	165 (100)	30 (100)	1.000
Meropenem	165 (100)	30 (100)	1.000
Imipenem	165 (100)	30 (100)	1.000
Cefepime	165 (100)	30 (100)	1.000
Piperacillin–tazobactam	165 (100)	30 (100)	1.000
Cefoperazone–sulbactam	120 (72.7)	18 (60.0)	0.191
Ampicillin–sulbactam	143 (86.7)	22 (73.3)	0.094
Polymyxin B	0 (0)	0 (0)	1.000
Colistin sulfate	0 (0)	0 (0)	1.000
Doxycycline	165 (100)	30 (100)	1.000

**Table 4:** Biofilm production and motility of MDR *A. baumannii* isolated from nonmedical devices and medical devices.

Source of isolate	Biofilm production				Twitching motility			Swarming motility	
	*n* (%)				*n* (%)			*n* (%)	
	None	Weak	Moderate	Strong	None	Intermediate	High	Negative	Positive
Nonmedical devices (tissues) (*n* = 165)	3 (2)	2 (1)	28 (17)	132 (80)	93 (56)	66 (40)	6 (4)	57 (35)	108 (65)
Medical devices (*n* = 30)	0 (0)	0 (0)	0 (0)	30 (100)	20 (67)	8 (27)	2 (7)	9 (30)	21 (70)
Total = 195	3	2	28	162	113	74	8	66	129

**Table 5:** Motility pattern of MDR *A. baumannii* showing resistance to different antibiotics.

Antibiotics	Number of MDR isolates showing resistance	Percent of resistant isolates				
		Twitching motility			Swarming motility	
		None	Intermediate	High	Negative	Positive
Cotrimoxazole	187	58.3	37.4	4.3	33.2	66.8
Gentamicin	190	57.4	38.4	4.2	33.7	66.3
Amikacin	184	56.0	39.7	4.3	34.2	65.8
Ciprofloxacin	193	58.0	37.8	4.2	34.2	65.8
Levofloxacin	181	56.4	39.2	4.4	34.8	65.2
Ceftazidime	195	58.0	38.0	4.0	33.8	66.2
Meropenem	195	58.0	38.0	4.0	33.8	66.2
Imipenem	195	58.0	38.0	4.0	33.8	66.2
Cefepime	195	58.0	38.0	4.0	33.8	66.2
Piperacillin–tazobactam	195	58.0	38.0	4.0	33.8	66.2
Cefoperazone–sulbactam	138	52.2	45.7	2.1	34.0	66.0
Ampicillin–sulbactam	165	57.0	40.6	2.4	35.8	64.2
Doxycycline	195	58.0	38.0	4.0	33.8	66.2

**Table 6:** Distribution of biofilm formation with motility in MDR *A. baumannii* isolates.

Biofilm types	Number (%)	Twitching motility			*p* value	Swarming motility		*p* value
		None	Intermediate	High		Negative	Positive	
Nonproducer	3 (1.5)	0	3 (1.5)	0	0.029	2 (1.0)	1 (0.5)	< 0.001
Weak producer	2 (1.0)	0	2 (1.0)	0		1 (0.5)	1 (0.5)	
Moderate producer	28 (14.3)	13 (6.7)	15 (7.7)	0		20 (10.2)	8 (4.1)	
Strong producer	162 (83.0)	100 (51.3)	54 (27.7)	8 (4.1)		43 (22.0)	119 (61.0)	

**Table 7:** Distribution of biofilm-related genes among 30 MDR *A. baumannii* isolates from medical devices.

Medical devices	*csuE* gene	*ompA* gene	*bap* gene	*bla*_PER−1_ gene
Endotracheal tube	15 (50.0%)	6 (20.0%)	6 (20.0%)	3 (10.0%)
Central venous catheter	7 (23.3%)	6 (20.0%)	5 (16.6%)	4 (13.3%)
Urinary catheter	5 (16.6%)	3 (10.0%)	3 (10.0%)	1 (3.3%)
CSF shunt	0.0%	1 (3.3%)	0.0%	0.0%

**Table 8:** Distribution of biofilm-related genes in *A. baumannii* isolates obtained from medical devices (*n* = 30) that exhibited resistance to different antibiotics.

Antibiotics	*csuE* (*n* = 27)	*ompA* (*n* = 16)	*bap* (*n* = 14)	*bla*_PER−1_ (*n* = 8)
Cotrimoxazole	27	16	14	8
Gentamicin	27	16	14	8
Amikacin	27	16	14	8
Ciprofloxacin	27	16	14	8
Levofloxacin	27	16	14	8
Ceftazidime	27	16	14	8
Cefepime	27	16	14	8
Piperacillin–tazobactam	27	16	14	8
Meropenem	27	16	14	8
Imipenem	27	16	14	8
Doxycycline	27	16	14	8
Cefoperazone–sulbactam	24	13	13	8
Ampicillin–sulbactam	25	14	13	8

## Data Availability

The data that support the findings of this study are available from the corresponding authors upon reasonable request.

## Nomenclature

*A. baumannii*: *Acinetobacter baumannii*
AHLs: Acyl homoserine lactones
ASM: American Society for Microbiology
ATCC: American Type Culture Collection
Bap: Biofilm-associated protein
BHI: Brain heart infusion
*bla*_PER−1_: beta-lactamase PER-1
CAUTI: Catheter-associated urinary tract infection
CLABSI: Central line–associated bloodstream infection
CTAB: Cetyltrimethylammonium bromide
CLSI: Clinical and Laboratory Standards Institute
COVID-19: Coronavirus disease 2019
DMSO: Dimethyl sulfoxide
EO: Essential oil
EDTA: Ethylenediaminetetraacetic acid
XDR: Extensively drug-resistant
FW: Forward primer
GC–MS: Gas chromatography–mass spectrophotometer
ICU: Intensive care unit
LB: Luria–Bertani
MIC: Minimum inhibitory concentration
MDR: Multidrug-resistant
NHRC: Nepal Health Research Council
OD: Optical density
OmpA: Outer membrane protein A
PCR: Polymerase chain reaction
RV: Reverse primer
TUTH: Tribhuvan University Teaching Hospital
VAP: Ventilator-associated pneumonia

## Supporting Information

Supporting Figure 1 (SF1): Motility exhibited by *A. baumannii* clinical isolates. Supporting Figure 2 (SF2): Examples of PCR products of the four biofilm-related genes in strains of *A. baumannii*. Supporting Figure 3 (SF3): Total ion chromatogram obtained by GC–MS of the essential oil of ginger. Supporting Figure 4 (SF4): Total ion chromatogram obtained by GC–MS of the essential oil of garlic. Supporting Figure 5 (SF5): Total ion chromatogram obtained by GC–MS of the essential oil of turmeric. Supporting Figure 6 (SF6): Total ion chromatogram obtained by GC–MS of the essential oil of *Ageratina adenophora.* Supporting Table 1 (ST1): Compound identified in the essential oil of ginger by GC–MS analysis. Supporting Table 2 (ST2): Compound identified in the essential oil of garlic by GC–MS analysis. Supporting Table 3 (ST3): Compound identified in the essential oil of turmeric by GC–MS analysis. Supporting Table 4 (ST4): Compound identified in the essential oil of *Ageratina adenophora* by GC–MS analysis.
