# Supplementary material for: Characterization and Biofilm Inhibition of Multidrug-Resistant Acinetobacter baumannii Isolates
Source: Int J Microbiol. 2024 Dec 28;2024:5749982. doi: 10.1155/ijm/5749982 (PMC11699987; doi:10.1155/ijm/5749982)
Supplement: Supporting Information — Additional supporting information can be found online in the Supporting Information section. [file 5749982.f1.docx]

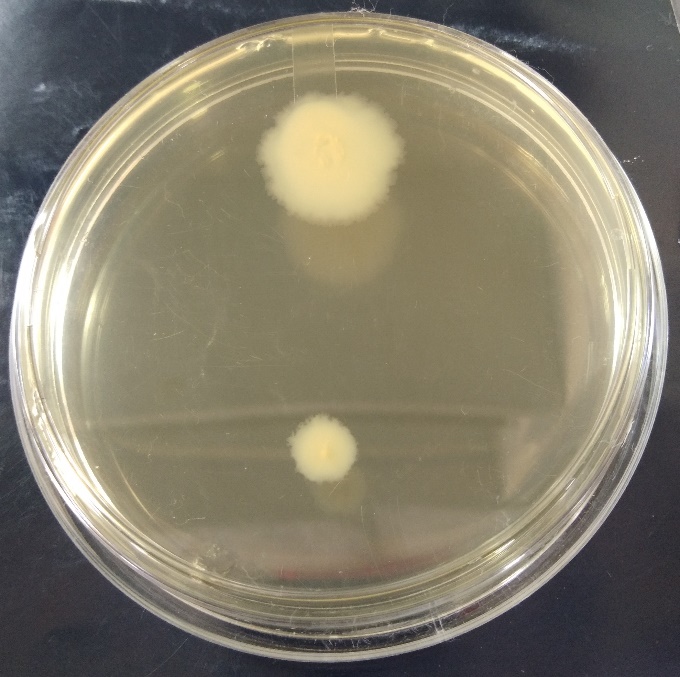

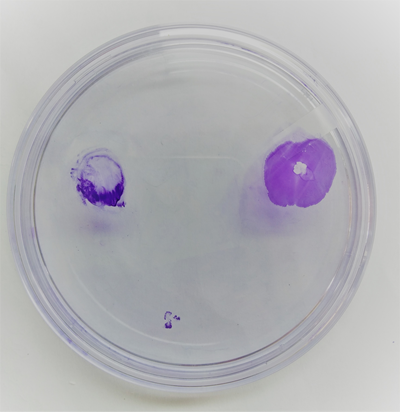


**Swarming Motility**

**Intermediately Motile**

**Non-Motile**

**Non-Motile**

**Highly Motile**

**B**

**A**

**SF1:** Motility exhibited by *A. baumannii* clinical isolates. **(A)** Showing swarming motility or being non-motile on 0.4% agar plates. **(B)** Showing twitching motility or being non-motile.


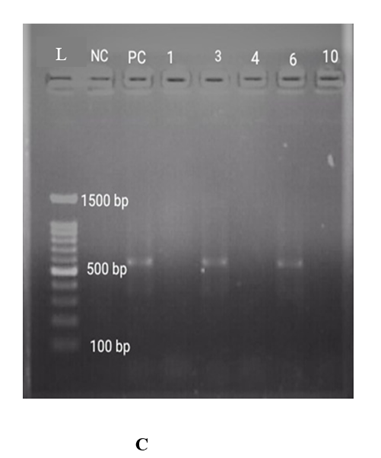

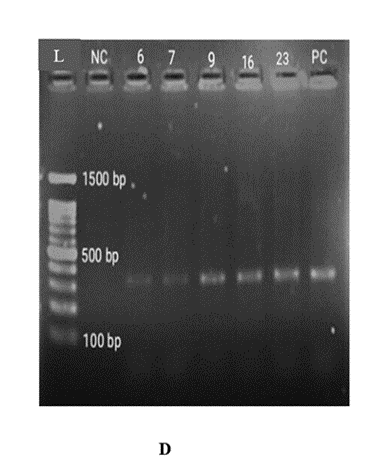

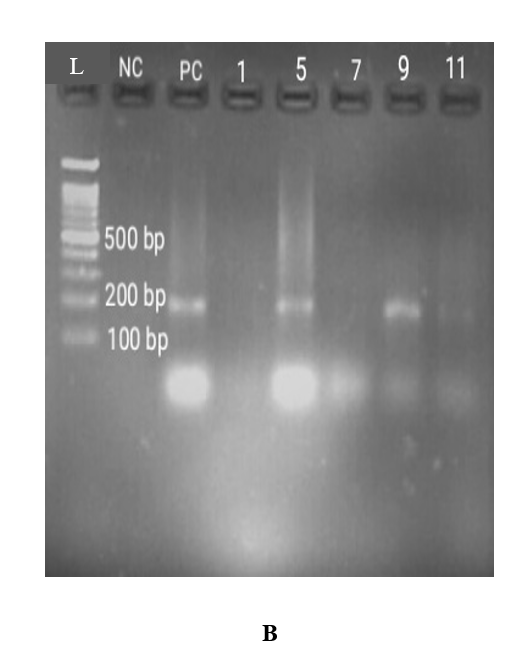

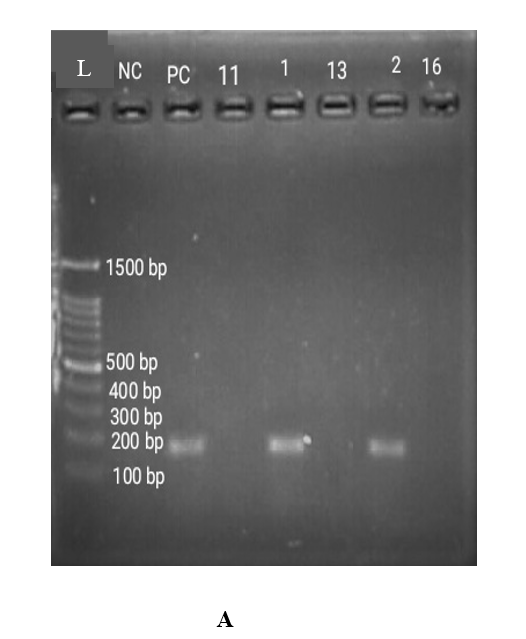


**SF2:** Examples of PCR products of the four biofilm-related genes in strains of *A. baumannii*. L = DNA ladder, NC= negative control, PC = positive control, numbers refer to different isolates of *A. baumannii*. **A:** *CsuE* gene, **B:** *bap* gene, **C:** *OmpA* gene, **D:** *blaPER-1* gene.

**SF3:** Total ion chromatogram obtained by GC-MS of the essential oil of ginger

**SF4:** Total ion chromatogram obtained by GC-MS of the essential oil of garlic

**SF5:** Total ion chromatogram obtained by GC-MS of the essential oil of turmeric

**SF6:** Total ion chromatogram obtained by GC-MS of the essential oil of *Ageratina adenophora*

**ST1:** Compound identified in the essential oil of ginger by GC-MS analysis

| **Peak No.** | **Retention Time** | **Compounds Name** | **Area %** |  | **Peak No.** | **Retention Time** | **Compounds Name** | **Area %** |
| --- | --- | --- | --- | --- | --- | --- | --- | --- |
| 1. | 13.247 | *α*-Pinene | 2.36 |  | 17. | 37.620 | Amorpha-4, 11-diene | 0.51 |
| 2. | 13.932 | Camphene | 6.78 |  | 18. | 38.278 | α-Selinene | 0.89 |
| 3. | 15.522 | 6-methyl-Hep-5-en-2-one | 0.59 |  | 19. | 38.397 | *α*-Curcumene | 6.84 |
| 4. | 15.689 | Myrcene | 1.34 |  | 20. | 38.923 | *β*-Curcumene | 14.86 |
| 5. | 17.616 | *β-*Phellandrene | 4.61 |  | 21. | 39.145 | *α*-Zingiberene | 2.38 |
| 6. | 17.744 | Eucalyptol | 2.26 |  | 22. | 39.301 | *α-*E,E-Farnesene | 8.30 |
| 7. | 20.892 | Linalool | 1.51 |  | 23. | 39.457 | *β*-Bisabolene | 5.66 |
| 8. | 24.316 | Borneol | 2.28 |  | 24. | 40.113 | *β-*Sesquiphellandrene | 10.28 |
| 9. | 25.437 | *α*-Terpineol | 0.63 |  | 25. | 41.545 | E-Nerolidol | 1.36 |
| 10. | 27.018 | Citronellol | 2.02 |  | 26. | 43.643 | Z,Z-Geranyl linalool | 0.92 |
| 11. | 27.736 | Neral | 5.74 |  | 27. | 44.300 | Himachalol | 0.78 |
| 12. | 28.270 | Geraniol | 3.33 |  | 28. | 45.329 | *β*-Eudesmol | 0.83 |
| 13. | 29.090 | Geranial | 9.00 |  | 29. | 45.414 | Bulnesol | 0.59 |
| 14. | 30.032 | Undecan-2-one | 0.89 |  | 30. | 46.461 | *α-*trans-E-Bergamotal | 0.74 |
| 15. | 34.010 | *α*- Copaene | 0.51 |  | 31. | 46.783 | *α*-trans-Z-Bergamotol | 0.58 |
| 16. | 37.163 | *β-*E-Farnesene | 0.62 |  |  |  |  |  |

**ST2:** Compound identified in the essential oil of garlic by GC-MS analysis

| **Peak No.** | **Retention Time** | **Compounds Name** | **Area %** |  | **Peak No.** | **Retention Time** | **Compounds Name** | **Area %** |
| --- | --- | --- | --- | --- | --- | --- | --- | --- |
| 1. | 10.159 | Diallyl-Sulfide | 0.93 |  | 8. | 23.020 | Allyl-methyl-trisulfide | 18.61 |
| 2. | 12.472 | Allyl-methyl- disulfide | 4.43 |  | 9. | 25.414 | 1H-Pyrrole, 1-methyl | 0.73 |
| 3. | 13.476 | 1,4-Dithiacyclohexane | 0.79 |  | 10. | 26.595 | Hydrazine, methyl | 2.20 |
| 4. | 14.915 | Dimethyl- trisulfide | 2.36 |  | 11. | 30.719 | Allitridin | 35.85 |
| 5. | 20.067 | Allyl-disulfide | 16.34 |  | 12. | 31.806 | Benzoic acid, 2-methyl | 2.32 |
| 6. | 20.772 | Allyl-trisulfide | 3.87 |  | 13. | 34.306 | 3-Vinyl-1,2-dithiacyclohex-5-en | 0.89 |
| 7. | 21.067 | Trisulfide-di-2 propenyl | 7.53 |  | 14. | 40.992 | Diallyl-tetrasulfide | 3.14 |

**ST3:** Compound identified in the essential oil of turmeric by GC-MS analysis

| **Peak No.** | **Retention Time** | **Compounds Name** | **Area %** |  | **Peak No.** | **Retention Time** | **Compounds Name** | **Area %** |
| --- | --- | --- | --- | --- | --- | --- | --- | --- |
| 1. | 16.404 | *α*-Phellandrene | 1.75 |  | 15. | 43.630 | *α*-Bisabolol | 0.62 |
| 2. | 17.349 | *p*-Cymene | 0.45 |  | 16. | 44.108 | Benzene, (1,1,4,6,6-pentamethylheptyl | 0.74 |
| 3. | 17.745 | Eucalyptol | 0.66 |  | 17. | 44.285 | Himachalol | 0.68 |
| 4. | 35.981 | E- Caryophyllene | 0.80 |  | 18. | 44.448 | 6-methly-2-(4-methylcyclohex-3-en-1-yl)hept-5-en-2-ol | 0.67 |
| 5. | 38.378 | *α*-Curcumene | 3.88 |  | 19. | 44.583 | Benzenamine, N-methyl-N-n-octyl | 0.71 |
| 6. | 38.888 | *α*-Zingiberene | 5.66 |  | 20. | 45.069 | Cis-Carvyl tiglate | 0.54 |
| 7. | 39.436 | *β-* Bisaboene | 1.11 |  | 21. | 45.401 | E-gamma-Atlantone | 1.34 |
| 8. | 40.087 | *β-* Sesquiphellandrene | 5.53 |  | 22. | 45.633 | ar-Tumerone | 19.89 |
| 9. | 40.230 | Nootkatene | 0.43 |  | 23. | 45.806 | Z-gamma-Atlantone | 29.02 |
| 10. | 40.423 | E-Gamma-Bisabolene | 0.54 |  | 24. | 47.021 | (E)-gamma-Atlantone | 18.06 |
| 11. | 42.214 | Curlone | 0.54 |  | 25. | 48.637 | Zinc, bis[2-(1,1-dimethyl-2-propenyl)-3,3-dimethyl | 0.56 |
| 12. | 42.292 | ar-Tumerol | 0.73 |  | 26. | 49.057 | Perilla alcohol tiglate | 0.48 |
| 13. | 42.740 | Fokienol | 0.54 |  | 27. | 49.629 | trans,α-Atlantone | 1.50 |
| 14. | 43.308 | ar-dihydro-Tumerone | 2.56 |  |  |  |  |  |

**ST4:** Compound identified in the essential oil of *Ageratina adenophora* by GC-MS analysis

| **Peak No.** | **Retention Time** | **Compounds Name** | **Area %** |  | **Peak No.** | **Retention Time** | **Compounds Name** | **Area %** |
| --- | --- | --- | --- | --- | --- | --- | --- | --- |
| 1. | 13.901 | Camphene | 3.52 |  | 21. | 40.072 | *β-* Sesquiphellandrene | 2.69 |
| 2. | 16.216 | *α-*Terpinene | 2.86 |  | 22. | 40.339 | *α*-methyl-Cinnamic alcohol | 0.66 |
| 3. | 16.383 | *α-*Phellandrene | 2.34 |  | 23. | 40.473 | Gamma-Vetivenene | 1.28 |
| 4. | 17.332 | *P-*Cymene | 4.77 |  | 24. | 40.764 | *α*-E- Bisabolene | 1.07 |
| 5. | 17.535 | Limonene | 0.77 |  | 25. | 41.211 | β- Vetivenene | 1.07 |
| 6. | 24.287 | Borneol | 0.87 |  | 26. | 43.621 | Z,Z-Geranyl linalool | 0.88 |
| 7. | 29.879 | Bornyl acetate | 5.82 |  | 27. | 44.172 | Khusilal | 3.44 |
| 8. | 35.963 | E-Caryophyllene | 1.42 |  | 28. | 44.282 | Hinesol | 1.47 |
| 9. | 36.438 | *α*-trans-Bergamotene | 1.47 |  | 29. | 44.637 | *α*-muurolol | 12.01 |
| 10. | 36.726 | Sesquisabinene | 0.73 |  | 30. | 44.824 | *α*- Eudesmol | 0.61 |
| 11. | 37.140 | *β*-E-Farnesene | 3.66 |  | 31. | 45.379 | Cadin-4-en-10-ol | 0.67 |
| 12. | 37.590 | Amorpha-4, 11-diene | 0.66 |  | 32. | 45.594 | Epizonarene | 4.04 |
| 13. | 38.236 | Gamma-Curcumene | 1.93 |  | 33. | 45.756 | Z-gamma-Atlantone | 3.92 |
| 14. | 38.382 | Epi-Zonarene | 3.89 |  | 34. | 46.383 | *α-* Bisabolol | 8.31 |
| 15. | 38.545 | D- Germacrene | 4.06 |  | 35. | 46.746 | Eremophilone | 1.20 |
| 16. | 38.654 | *β*- Chamigrene | 0.92 |  | 36. | 47.032 | Cyperotundone | 6.42 |
| 17. | 38.869 | Nerolidol | 0.89 |  | 37. | 47.998 | *p-*Mentha-1,4(8)-diene | 1.08 |
| 18. | 39.176 | Bicyclogermacrene | 1.50 |  | 38. | 48.805 | 13-hydroxy-valencene | 0.73 |
| 19. | 39.425 | *β*- Bisabolene | 4.54 |  | 39. | 53.264 | Zederone | 1.13 |
| 20. | 39.773 | Gamma, Z- Bisabolene | 0.69 |  |  |  |  |  |
